# Evaluation of the Levels of Selected Cytokines and Their Possible Influence on the Development of Cardiovascular and Pulmonary Complications in Patients after COVID-19

**DOI:** 10.3390/medicina60030353

**Published:** 2024-02-21

**Authors:** Anita Stanjek-Cichoracka, Jacek T. Niedziela, Anna Łaszewska, Zofia Mędrala, Alicja Nowowiejska-Wiewióra, Jacek Kaczmarski, Alicja Grzanka, Mariusz Gąsior

**Affiliations:** 1 Department of Biophysics, Faculty of Pharmaceutical Sciences in Sosnowiec, Medical University of Silesia in Katowice, Jedności 8, 41-200 Sosnowiec, Poland; 2 Laboratory of Transplant Immunology, Silesian Centre for Heart Diseases, 41-800 Zabrze, Poland; alaszewska@sccs.pl; 3 3rd Department of Cardiology, Silesian Center for Heart Disease, 41-800 Zabrze, Poland; jniedziela@sum.edu.pl (J.T.N.); zmedrala@sccs.pl (Z.M.); anowowiejska-wiewiora@sccs.pl (A.N.-W.); mgasior@sum.edu.pl (M.G.); 4 3rd Department of Cardiology, Faculty of Medical Sciences in Zabrze, Medical University of Silesia, 40-752 Katowice, Poland; 5 Department of Laboratory Diagnostics of Environmental and Civilization Diseases, Section of Coagulation Disorders, Silesian Center for Heart Disease, 41-800 Zabrze, Poland; jkaczmarski@sccs.pl; 6 Department of Allergology, Faculty of Medical Sciences in Zabrze, Medical University of Silesia, 40-752 Katowice, Poland; agrzanka@sum.edu.pl

**Keywords:** cytokines, COVID-19, cardiovascular and pulmonary complications, Luminex

## Abstract

*Background and Objectives*: The aim of this study was to evaluate the levels of selected cytokines and their possible influence on the development of cardiovascular and pulmonary complications in patients hospitalized at the Silesian Centre for Heart Disease in Zabrze after having undergone COVID-19. *Materials and methods*: The study included 76 randomly selected patients from the SILCOVID-19 database. The median time from symptom onset to the study visit was 102 (86–118) days. The median age of the study group was 53 (44–60) years. Assays of a panel of 30 cytokines were carried out in the serum of patients on a Luminex100 platform using the Milliplex MAP kit from Merck KGaA Germany. *Results*: There were no statistically significant differences in most of the cytokines analyzed between patients with confirmed or excluded lung lesions or cardiac abnormalities. Additionally, no statistically significant differences in cytokine concentrations according to gender, age, comorbidity of diabetes, renal disease, hypertension, increased risk of thrombotic disease, or psychological disorders were demonstrated. There were high concentrations of cytokines such as platelet-derived growth actor-AA (PDGF-AA), monocyte chemoattractant protein-1 (MCP-1), monokine-induced gamma interferon (MIG), and vascular endothelial growth factor-A (VEGF-A). *Conclusions:* No direct impact of the dependencies between a panel of cytokines and the incidence of cardiovascular and pulmonary complications in patients hospitalized at the Silesian Centre for Heart Disease in Zabrze after having undergone COVID-19 was demonstrated. The demonstration of high levels of certain cytokines (PDGF-AA, VEGF, MIG, and IP10) that are of significance in the development of many lung diseases, as well as cytokines (MCP-1) that influence the aetiopathogenesis of cardiovascular diseases seems to be highly concerning in COVID-19 survivors. This group of patients should receive further monitoring of these cytokine levels and diagnostic imaging in order to detect more severe abnormalities as early as possible and administer appropriate therapy.

## 1. Introduction

The COVID-19 pandemic, its aetiopathogenesis, the risk factors influencing the course of the disease, and the development of complications have been the subject of much research. This research includes the spectrum of clinical symptoms that appear in a given patient and its dependence on genetic factors, the proper functioning of the immune system, and the presence of comorbidities [1,2].

In the available literature, there are many papers on the evaluation of the profile of different cytokines in the acute phase of COVID-19. These studies have shown that inflammatory biomarker levels are elevated in patients with COVID-19 and that higher levels of inflammatory cytokines are consistently associated with a more severe disease course and poorer treatment outcomes. This data has sparked interest in the “cytokine storm” as a major factor influencing disease severity in COVID-19 [3,4]. 

Once the acute phase of the disease is over, some patients experience symptoms that may persist for months, referred to as long COVID. This condition affects the functioning of many organs, but its causes are not fully understood. Patients with symptomatic COVID-19 have an excessive inflammatory response in the acute phase, and this perhaps represents a potential cause, affecting immune cell function even after recovery [5]. 

“Long COVID” can be defined as the signs, symptoms, and sequelae that continue or develop after acute COVID-19 or SARS-CoV-2 infection for any period; they are generally multisystemic and might present with a relapsing–remitting pattern and a progression or worsening over time, with the possibility of severe and life-threatening events even months or years after infection [6].

In the long-term follow-up of post-COVID complications, the fibrotic lesions observed in the lungs appear to be of particular importance.

These processes appear to be closely related to the severity of the course of COVID-19, genetic and idiopathic factors, the presence of chronic diseases, age, and sex [7,8].

The available literature shows that “long COVID” also contributes to cardiovascular dysfunction, causing cardiac arrhythmias, thromboembolic disorders, myocarditis, and ischemic heart disease, among others. The risk of these diseases is increased in people with severe COVID-19 and those with comorbidities such as hypertension, kidney disease, obesity, hyperlipidemia, and diabetes [9].

Assessing the cytokine profile can also help in identifying markers of future health problems and EQQ, thus assisting in planning an appropriate treatment plan and preventing long-term health effects in patients after COVID-19.

The aim of this study was to evaluate the levels of selected cytokines and their possible influence on the development of cardiovascular and pulmonary complications in patients hospitalized at the Silesian Centre for Heart Disease in Zabrze after having undergone COVID-19.

## 2. Materials and Methods

This Silesian study on COVID-19 complications (SILCOV-19) was a prospective observational registry-based study focusing on the complications after COVID-19 in the Silesian population in Poland. Two hundred adult patients with confirmed symptomatic SARS-CoV-2 infection in the past were enrolled between June 2020 and March 2021 in the third department of Cardiology at the Silesian Centre for Heart Disease in Zabrze, the Faculty of Medical Sciences in Zabrze and the Medical University of Silesia in Katowice. The following inclusion criteria were used: (1) age of 18 years or more, (2) SARS-CoV-2 RNA confirmed via a polymerase chain reaction (PCR) in the acute phase of the disease, (3) the presence of the clinical symptoms associated with COVID-19 in the acute phase of the disease, and (4) two negative SARS-CoV-2 PCR test results following a seven-day period of quarantine after symptom regression. The exclusion criteria included a lack of patient’s informed consent. General information on the SILCOV-19 database was reported previously [10]. The study included 76 randomly selected patients from the SILCOV-19 database. The median age of the study group was 53 (44–60) years. The median time from symptom onset to the study visit was 102 (86–118) days.

From each patient, 2.7 mL of whole blood was collected into test tubes and centrifuged at 300 g for 10 min at 25 °C to separate the blood cells and serum. The serum obtained was frozen and stored at −80 °C. Portions of serum were thawed immediately before the analysis.

Assays of a panel of 30 cytokines were carried out on a Luminex100 platform using the Milliplex MAP Kit from Merck KGaA, Darmstadt, Germany.

The human cytokine/chemokine/growth factor panel A bead-based multiplex panel, using Luminex^®^ xMAP technology (Luminex Corporation, Austin, TX, USA), enables the simultaneous analysis of multiple cytokines, chemokines, and growth factor biomarkers in human serum, plasma, and cell culture samples. This is an overnight or two-hour incubation assay. This assay requires 25 μL of neat plasma or serum or 25 μL of cell culture supernatant per well.

Luminex xMAP technology is one of the fastest growing and most respected multiplex technologies offering applications throughout the life sciences and capable of performing a variety of bioassays, including immunoassays on the surface of fluorescent-coded magnetic beads known as MagPlex-c microspheres [11].

The selected panel of cytokines allowed for the simultaneous determination of concentrations of 30 analytes: fibroblast growth factor 2 (FGF-2), granulocyte colony-stimulating factor (G-CSF), granulocyte-macrophage colony-stimulating factor (GM-CSF), interferon alpha-2 (IFN-α2), interferon gamma (IFN-γ), interleukin 1 beta (IL-1β), interleukin-1 receptor antagonist protein (IL-1ra), interleukin 2 (IL-2), interleukin 4 (IL-4), interleukin 5 (IL-5), interleukin 6 (IL-6), interleukin 7 (IL-7), interleukin 8 (IL-8), interleukin 9 (IL-9), interleukin 10 (IL-10), interleukin-12 subunit p40 (IL-12 (p40)), interleukin-12 subunit p 70 (IL-12 (p70)), interleukin 13 (IL-13), interleukin 15 (IL-15), interleukin *17E* (*IL*-*17E*), also known as *IL*-*25* (IL-17E/IL25), interleukin 17F (IL-17F), interleukin 18 (IL18), interferon gamma-induced protein *10* (IP-10), monocyte chemoattractant protein-1 (MCP-1), monokine-induced gamma interferon (MIG), macrophage inflammatory protein-1 alpha (MIP-1α), macrophage inflammatory protein-1alpha (MIP-1β), platelet-derived growth factor-AA (PDGF-AA), tumor necrosis factor alfa (TNF-α), and vascular endothelial growth factor-A (VEGF-A).

The local bioethical committee approved the study (approval No. 17/2020 dated 1 June 2020). The study was registered on ClinicalTrials.gov (NCT04453748, https://clinicaltrials.gov/ct2/show/NCT04453748, accessed on 8 June 2020). The study was performed under the patronage of the Polish Cardiac Society.

### Statistical Analysis

The normality of the variables was evaluated using the Shapiro–Wilk test. All continuous variables had a non-normal distribution and were presented as a median (interquartile range [IQR]). The categorical variables were shown as percentages. The patients with normal (≤5 mg/dL) and elevated (>5 mg/dL) levels of high-sensitivity C-reactive protein (hsCRP) were compared using the nonparametric Mann–Whitney test for the continuous variables without a normal distribution, and the χ^2^ test for the categorical data with Yates correction if applicable. The statistical significance was defined as *p* < 0.05. All statistical analyses were performed using TIBCO Statistica v.13.3.0 (TIBCO Software Inc., Palo Alto, CA, USA)

## 3. Results

The data from the SILCOV database mentioned earlier was used for a complete analysis of the obtained results. The baseline characteristics are shown in Table 1.

Table 2 and Table 3 both show the results of the basic laboratory tests of the study group.

The selected panel of cytokines allowed for the simultaneous determination of concentrations of 30 analytes. However, in further statistical analysis, those proteins whose concentrations were below the sensitivity of the method and the range of the lowest standard of the standard curve were omitted. The concentrations of the following cytokines were analyzed: fibroblast growth factor 2 (FGF-2), interleukin-1 receptor antagonist protein (IL-1ra), interleukin 5 (IL-5), interleukin 6 (IL-6), interleukin 8 (IL-8), interleukin 9 (IL-9), interleukin 10 (IL-10), interleukin-12 subunit p40 (IL-12 (p40)), interleukin 15 (IL-15), interleukin 18 (IL18), interferon gamma-induced protein *10* (IP-10), monocyte chemoattractant protein-1 (MCP-1), monokine-induced gamma interferon (MIG), macrophage inflammatory protein-1 beta (MIP-1β), platelet-derived growth factor-AA (PDGF-AA), tumor necrosis factor alfa (TNF-α), and vascular endothelial growth factor-A (VEGF-A).

The cytokine results for the entire study group are shown in Table 4.

No statistically significant differences in cytokine concentrations according to gender, age (above and below the median), comorbidity of diabetes, renal disease, hypertension, or an increased risk of thrombotic disease were demonstrated.

The study group was also divided according to the high-sensitivity C-reactive protein (hsCRP) results (≤5 mg/dL and >5 mg/dL). The results are shown in Table 5.

The only statistically significant finding was the lower concentration of interferon gamma-induced protein 10 (IP-10) in the group with elevated hsCRP values.

When analyzing the possible complications after COVID-19, the study group was divided according to the values of N-terminal pro-Btype natriuretic peptide (NTproBNP) concentrations (≤125 pg/mL and >125 pg/mL). The results are shown in Table 6.

Statistically significant lower levels of IL-8 and monocyte chemoattractant protein-1 (MCP-1) were found in the group with elevated NTproBNP levels.

In all the patients, left ventricular function was assessed via echocardiography using the global longitudinal strain (GLS) parameter.

The study group was divided according to GLS values ≤ 18 and >18 (Table 7).

Statistically significant higher levels of IL-18, monokine-induced gamma interferon (MIG), and vascular endothelial growth factor-A (VEGF-A) were found in the group of patients with abnormal GLS values.

All of the patients underwent high-resolution computed tomography (HRCT). The analysis of the HRCT results showed the appearance of lesions in lung imaging in 22 patients. In this group, the only statistically lower concentrations were those of macrophage inflammatory protein-1 beta (MIP-1b) and tumor necrosis factor-α human (TNF-α) (Table 8).

When analyzing the concentrations of pro-inflammatory cytokines associated with a “cytokine storm” (IL-1β, IL-1RA, IL-6, IL-8, IL-18, and TNF-a) and cytokines that inhibit these processes (IL-10 and IL-1RA) in both the whole group and the patient subdivisions shown above, no statistically significant differences in their concentrations were noted.

An important value of these studies is the demonstration of high concentrations of cytokines, such as PDGF -AA, MCP-1b, MIG, and VEGF-A, in the whole study group, which may be related to the possible development of further post-COVID complications.

## 4. Discussion

The so-called “cytokine storm” in the course of COVID-19 infection has been the subject of research conducted by many scientists around the world. It has become important to determine the cytokine profile in active infection.

According to the literature available, changes in cytokine concentrations (especially pro-inflammatory cytokines) relate to patients with confirmed SARS-CoV2 infection versus controls without that pathogen [12]. Some publications have confirmed that an appropriately selected cytokine profile can predict the development of acute respiratory distress syndrome (ARDS) or acute kidney injury [13].

However, studies by Wilson et al. showed that inflammatory cytokine levels were not significantly higher in patients with severe or moderate COVID-19 or critically ill patients with ARDS or sepsis [1].

Another focus of the study was an attempt to answer the question of how cytokines behave in patients after undergoing SARS-CoV-2 infection and whether they have an impact on the development of complications.

In the available literature, it is possible to find data showing that “long COVID” symptoms were reported by up to 80% of patients, irrespective of the severity of the disease, but were more common in patients who required hospitalization [14].

In a study by Santopaulo et al., patients with a severe course of COVID-19 reported a higher number of long-term symptoms, which, however, did not correlate with pro-inflammatory cytokines [15].

In contrast, Ruenjaiman et al. found that SARS-CoV-2 infection had a lasting effect on immunity at the cellular level as well as on cytokine production levels [16].

In our study, there were no statistically significant differences in most of the cytokines analyzed between the patients with confirmed or excluded lung lesions (based on HRCT) or cardiac function (GLS or NTproBNP testing).

In our study, the results of the cardiovascular tests did not reveal any severe complications. Only 18% of the study group had elevated NT BNP values and demonstrated GLS changes, which may indicate the development of heart failure.

On the basis of HRCT, it was shown that 28% of the studied patients had fibrotic lesions in their lungs.

Data on functional or radiological long-term complications are currently available in hospitalized individuals with COVID-19 pneumonia or SARS [17,18].

Additionally, no statistically significant differences in cytokine concentrations according to gender, age, comorbidity of diabetes, renal disease, hypertension, increased risk of thrombotic disease, or psychological disorders were demonstrated.

However, the demonstration of high concentrations of PDGF-AA, MCP-1b, MIG, and VEGF-A, among others, is highly concerning.

One of the chemokines that was assayed in the patients of the study group and whose levels were high was MCP-1 (with a median of 297.147 pg/mL). It is a monocyte chemotactic protein that plays a key role in the pathogenesis of cardiovascular diseases. MCP-1, through its chemotactic activity, induces the diapedesis of monocytes and macrophages from the blood vessel lumen into the subendothelium, where foam cells are formed, resulting in atherosclerotic plaque formation. Macrophages, on the other hand, are responsible for plaque instability, which can result in an ischemic episode or recurrent restenosis after angioplasty. There are many reports on the function of MCP-1 in ischemia/reperfusion or rejection of the transplanted heart [19,20,21].

There were no statistically significant differences between MCP-1 levels and GLS changes or high NTproBNP values, but consideration should be given to extending the cardiovascular diagnosis of patients in the study group.

Our own studies have shown high concentrations of PDGF-AA. The median concentration in the study group was 2968.137 pg/mL.

PDGF-AA is a dimorphic isoform of platelet-derived growth factor, which regulates cell differentiation and division. It also plays an important role in angiogenesis. It has an important function in the pathogenesis of fibrosis. These isoforms promote the proliferation and chemotaxis of myofibroblasts [22,23].

PDGF is also a well-recognized factor mediating airway inflammation and remodeling in asthma. PDGF stimulates airway smooth muscle cell (ASMC) proliferation and ASMC migration into the epithelium, as well as increased collagen synthesis in the lungs. Most studies on PDGF in asthma have not suggested that this factor is a possible biomarker of disease severity [24].

However, it is this growth factor that is thought to be one of the main contributors to airway remodeling.

The importance of this remodeling in asthma is well acknowledged, and new therapies should also aim to deal with it [25,26,27].

In the long term, PDGF-AA should be monitored in the study group and complemented by imaging and functional tests, e.g., spirometry.

Our studies also showed high concentrations of VEGF-A (with a median of 177.832 pg/mL), MIG (with a median of 372.420 pg/mL), and IP10 (with a median of 61.698 pg/mL) in the whole group.

Vascular endothelial growth factor (VEGF) is a pluripotent growth and permeability factor that has broad effects on endothelial cell function. This protein is also associated with angiogenesis but is often treated as a marker of cancer development. Lung tissue is very abundant in this protein. Many different lung cells produce VEGF and respond to it. VEGF is crucial for lung development and serves as a sustaining factor for lung function in adulthood. In addition to the physiological functions of this protein, there is growing evidence that VEGF also plays a role in several acute and chronic lung diseases, such as acute lung injury, severe pulmonary hypertension, and emphysema [28,29].

MIG, also known as C-X-C motif chemokine ligand 9 (CXCL9), is an inflammatory chemokine and a key component of the inflammatory response, attracting primarily T lymphocytes and NK cells. MIG has angiostatic properties, plays an important role in the inflammatory response, and performs functions that may contribute to lung carcinogenesis. The overexpression of MIG observed prior to the diagnosis of lung cancer may also reflect compensatory efforts on the part of the immune system to prevent ongoing pro-angiogenic changes in the tissue microenvironment [30,31,32,33].

Related to MIG is also IP-10. IP-10 is an important mediator of the recruitment of activated lymphocytes to the lungs in lung diseases and is involved in the response to mycobacterium tuberculosis, among others. Data in the literature suggests that IP-10 is a potential marker for lung diseases. IP10 is recognized as a biomarker of human rhinovirus infection in COPD exacerbation, and the importance of this interleukin in pulmonary fibrosis is being investigated [26].

In their study, Tamayo-Velasco et al. further demonstrated the usefulness of IP-10 as an excellent marker in clinical practice for the diagnosis of COVID-19 in the hospital setting. According to these researchers, IP-10 can be used as a complementary tool in clinical practice, especially in emergency departments [12].

It seems that an appropriately selected panel of cytokines could provide a useful predictive tool to assess the occurrence of possible complications after COVID-19.

## 5. Conclusions

No direct impact of the dependencies between a panel of cytokines and the incidence of cardiovascular and pulmonary complications in patients hospitalized at the Silesian Centre for Heart Disease in Zabrze after having undergone COVID-19 was demonstrated. The demonstration of high levels of certain cytokines (PDGF-AA, VEGF, MIG, and IP10) that are of significance in the development of many lung diseases, as well as cytokines (MCP-1) that influence the aetiopathogenesis of cardiovascular diseases seems to be highly concerning in COVID-19 survivors. This group of patients should receive further monitoring of these cytokine levels and diagnostic imaging in order to detect more severe abnormalities as early as possible and administer appropriate therapy.

## Figures and Tables

**Table 1:** Baseline characteristics of the study group.

Medical History, Diseases before COVID-19	
Hypertension, *n* (%)	28 (36.8)
Hyperlipidemia, *n* (%)	22 (28.9)
Diabetes mellitus, *n* (%)	13 (17.1)
Smoking, *n* (%)	10 (13.2)
Coronary artery disease, *n* (%)	7 (9.2)
Percutaneous coronary intervention, *n* (%)	5 (6.6)
Myocardial infarction, *n* (%)	4 (5.3)
Asthma/COPD, *n* (%)	1 (1.3)
Chronic kidney disease, *n* (%)	1 (1.3)
Stroke, *n* (%)	1 (1.3)

Abbreviations: COPD, chronic obstructive pulmonary disease.

**Table 2:** Results of selected hematological and coagulological laboratory tests in the study group.

Parameters	N	Median	Q1	Q3
White blood cells [10^3^/mm^3^] N:4.3–10	76	6.1500	5.1400	7.5100
Neutrophils [10^3^/mm^3^] N:2.9–4.1	76	3.7650	2.9150	4.5250
Lymphocytes [10^3^/mm^3^] N:1.7–2.8	76	1.6900	1.4050	2.2700
Monocytes [10^3^/mm^3^] N:0.2–0.8	76	0.4900	0.4000	0.5500
Eozynofiles [10^3^/mm^3^] N:0.2–0.4	76	0.1000	0.0500	0.1600
Basophils [10^3^/mm^3^] N:0–0.1	76	0.0400	0.0250	0.0600
Red blood cells [10^6^/mm^3^] N:4.5–5.9	76	4.6050	4.3900	4.9250
Hemoglobin [mmol/L] N:8.7–11.2	76	8.6000	8.2000	9.1000
Hematocrit [L/L] N:41–53	76	41.0000	39.0000	43.0500
Platelets [10^3^/mm^3^] N:150–350	76	250.0000	211.0000	275.5000
Fibrinogen [mg/dL] N:200–400	76	327.5000	285.0000	375.5000
D-Dimer [ug/mL] N:0–0.5	76	0.3000	0.2700	0.3900

**Table 3:** Results of selected biochemical laboratory tests in the study group.

Parameters	N	Median	Q1	Q3
GGTP [U/l] N:5–61	76	23.0000	17.0000	36.5000
AST [U/l] N:10–34	76	21.0000	19.0000	25.0000
ALT[U/l] N:6–44	76	22.0000	16.5000	31.5000
ALP [U/l] (N:40–129)	76	65.0000	57.5000	76.0000
Bilirubin [µmoL/L]	76	10.4500	7.8000	12.8500
Creatinine [mg/dL]	76	0.8371	0.7014	0.9615
GFR [mL/min × 1.73 m^2^]	76	113.2665	87.7913	136.3501
Uric acid [mmol/L]	76	332.0000	281.0000	395.5000
Total protein in serum [g/L]	76	72.0000	69.5000	76.0000
Albumin [g/L]	76	47.0000	46.0000	49.0000
HbA1c [%]	76	5.5000	5.2500	5.9000
Total cholesterol [mmol/L]	76	5.3350	4.3900	6.1050
Triglycerides [mmol/L]	76	1.3900	0.8750	1.9100
HDL-chlesterol [mmol/L]	76	1.5600	1.2050	1.7950
LDL-cholesterol [mmol/L]	76	3.4950	2.6200	4.3900
hs CRP [mg/dL]	76	2.3600	0.4800	5.1900
NT-proBNP [pg/mL]	76	66.8300	37.2650	110.6000
CK [U/l]] (N:24–193)	76	105.5000	85.5000	163.0000
CK-MB [ng/mL] (N:0.3–4.87)	76	2.0600	1.6950	2.7300
Troponin T hs [ng/mL] (pg/mL) (N < 0.014)	76	6.0000	4.0000	8.0000
LDH [U/l] (N:135–225)	76	185.5000	165.0000	211.5000
Pancreatic amylase [U/l] (N:15–53)	76	25.0000	19.0000	32.0000
Ferritin [ng/mL] N:30–400	76	97.0000	55.5000	156.0000
Lactates	70	1.4850	1.2500	2.0000

Abbreviations: alanine aminotransferase (ALT), alkaline phosphatase (ALP), aspartate aminotransferase (AST), creatine phosphokinase (CK), creatine kinase myocardial band (CK-MB), gamma-glutamyl transpeptidase (GGTP), glomerular filtration rate (GFR), hemoglobin A 1c (HbA1c), high-sensitivity C-reactive protein (hs CRP), lactate dehydrogenase (LDH), N-terminal pro–B-type natriuretic peptide (NT-proBNP).

**Table 4:** Concentrations of the cytokines analyzed for the entire study group.

	Median [pg/mL]	Q1	Q3
FGF-2	44.215	25.600	94.637
IL-1RA	3.611	1.854	4.772
IL-5	0.602	0.335	0.858
IL-6	0.640	0.507	1.363
IL-8	6.099	4.359	8.733
IL-9	2.315	1.268	3.915
IL-10	2.793	1.481	3.821
IL-12 (p40)	18.637	9.198	24.900
IL-15	5.758	4.077	6.826
IL-18	9.343	5.934	13.375
IP-10	61.698	49.170	88.491
MCP-1	297.147	224.013	387.643
MIG	372.420	294.823	618.893
MIP-1b	23.820	17.378	35.380
PDGF-AA	2968.137	2313.470	3546.726
TNF a	8.005	5.401	12.343
VEGF-A	177.832	116.289	281.695

**Table 5:** Cytokine concentrations in patient groups in relation to the hsCRP values.

	hsCRP ≤ 5 n = 23			hsCRP > 5 n = 53			
	Median [pg/mL]	Q1	Q3	Median [pg/mL]	Q1	Q3	*p*
FGF-2	44.215	25.600	100.272	62.047	25.600	89.001	0.817
IL-1RA	3.611	1.854	5.437	3.364	1.600	4.102	0.216
IL-5	0.514	0.242	0.773	0.640	0.426	1.270	0.113
IL-6	0.770	0.552	1.446	0.640	0.418	1.027	0.100
IL-8	5.898	4.257	7.604	6.298	4.749	9.829	0.309
IL-9	2.221	1.387	3.915	2.944	1.148	4.366	0.557
IL-10	2.909	1.481	3.821	2.443	1.481	3.595	0.360
IL-12 (p40)	18.637	9.198	26.922	14.127	9.198	18.637	0.214
IL-15	5.758	4.077	7.348	5.210	4.077	6.297	0.112
IL-18	9.168	5.860	11.140	11.256	6.508	16.601	0.218
IP-10	66.072	55.094	96.447	53.658	39.555	70.131	0.009
MCP-1	289.025	230.827	358.730	312.395	217.318	427.562	0.627
MIG	405.711	294.231	639.089	347.579	295.414	550.366	0.203
MIP-1b	23.820	19.300	33.724	22.810	14.284	40.249	0.549
PDGF-AA	3066.426	2277.874	3607.844	2856.383	2407.816	3487.384	0.483
TNF-a	8.005	5.401	11.736	8.639	5.401	12.343	0.923
VEGF-A	179.501	126.740	280.661	170.798	94.738	283.023	0.557

**Table 6:** Cytokine results in patient groups according to NTproBNP levels.

	NTproBNP ≤ 125 pg/mL N = 62			NTproBNP > 125 pg/mL N = 14			
	Median [pg/mL]	Q1	Q3	Median [pg/mL]	Q1	Q3	*p*
FGF-2	44.215	25.600	89.001	69.260	25.600	129.368	0.312
IL-1RA	3.611	1.854	4.590	2.866	1.600	5.798	0.936
IL-5	0.602	0.426	1.107	0.514	0.242	0.688	0.359
IL-6	0.683	0.507	1.446	0.640	0.596	0.942	0.995
IL-8	6.369	4.692	9.015	4.372	3.470	5.870	0.006
IL-9	2.592	1.387	4.366	1.823	0.640	2.592	0.094
IL-10	2.909	1.481	3.821	2.502	1.481	3.821	0.825
IL-12 (p40)	16.422	9.198	26.922	18.637	9.198	20.785	0.979
IL-15	5.758	4.077	7.088	5.348	4.077	6.826	0.391
IL-18	9.154	6.008	12.929	9.604	5.477	16.486	0.989
IP-10	61.698	52.453	85.662	58.772	38.340	92.832	0.384
MCP-1	309.132	238.170	414.637	223.139	179.357	301.860	0.003
MIG	399.644	295.414	629.511	356.657	200.168	550.366	0.414
MIP-1b	24.191	19.157	36.614	19.317	14.284	27.608	0.053
PDGF-AA	3047.761	2425.039	3571.481	2479.284	2064.795	3487.384	0.152
TNF-a	8.005	5.401	12.343	7.685	5.401	16.504	0.920
VEGF-A	179.356	115.127	283.023	171.379	130.220	275.055	0.693

**Table 7:** Cytokine concentrations in patients in relation to the GLS values.

	NTproBNP ≤ 125 pg/mL N = 62			NTproBNP > 125 pg/mL N = 14			
	Median [pg/mL]	Q1	Q3	Median [pg/mL]	Q1	Q3	*p*
FGF-2	44.215	25.600	89.001	69.260	25.600	129.368	0.312
IL-1RA	3.611	1.854	4.590	2.866	1.600	5.798	0.936
IL-5	0.602	0.426	1.107	0.514	0.242	0.688	0.359
IL-6	0.683	0.507	1.446	0.640	0.596	0.942	0.995
IL-8	6.369	4.692	9.015	4.372	3.470	5.870	0.006
IL-9	2.592	1.387	4.366	1.823	0.640	2.592	0.094
IL-10	2.909	1.481	3.821	2.502	1.481	3.821	0.825
IL-12 (p40)	16.422	9.198	26.922	18.637	9.198	20.785	0.979
IL-15	5.758	4.077	7.088	5.348	4.077	6.826	0.391
IL-18	9.154	6.008	12.929	9.604	5.477	16.486	0.989
IP-10	61.698	52.453	85.662	58.772	38.340	92.832	0.384
MCP-1	309.132	238.170	414.637	223.139	179.357	301.860	0.003
MIG	399.644	295.414	629.511	356.657	200.168	550.366	0.414
MIP-1b	24.191	19.157	36.614	19.317	14.284	27.608	0.053
PDGF-AA	3047.761	2425.039	3571.481	2479.284	2064.795	3487.384	0.152
TNF-a	8.005	5.401	12.343	7.685	5.401	16.504	0.920
VEGF-A	179.356	115.127	283.023	171.379	130.220	275.055	0.693

**Table 8:** Cytokine concentrations in the patient groups in relation to the HRCT results.

	No Change in HRCT N = 53			Changes in HRCT N = 22			
	Median [pg/mL]	Q1	Q3	Median [pg/mL]	Q1	Q3	*p*
FGF-2	44.215	25.600	89.001	65.100	25.600	100.272	0.740
IL-1RA	3.364	1.854	4.590	3.611	1.854	4.954	0.889
IL-5	0.602	0.335	1.107	0.621	0.426	0.773	0.958
IL-6	0.640	0.507	1.363	0.662	0.507	1.446	0.917
IL-8	6.127	4.634	8.902	6.084	3.937	7.378	0.701
IL-9	2.221	1.148	3.603	3.194	1.387	5.354	0.314
IL-10	2.560	1.481	3.821	2.909	2.560	5.590	0.164
IL-12 (p40)	16.422	9.198	22.877	18.637	6.400	22.877	0.816
IL-15	5.758	4.077	6.826	5.758	3.784	6.826	0.749
IL-18	9.517	6.067	14.539	9.343	6.008	10.735	0.456
IP-10	62.496	52.483	85.662	60.284	48.106	92.524	0.820
MCP-1	305.242	238.170	412.309	271.486	191.706	355.065	0.093
MIG	429.751	303.664	614.843	356.374	252.034	835.108	0.545
MIP-1b	25.522	19.300	37.912	22.211	14.284	25.044	0.038
PDGF-AA	2913.182	2253.703	3419.289	3392.182	2652.544	3747.071	0.161
TNS-a	8.639	6.064	12.343	6.718	4.044	9.268	0.029
VEGF-A	179.210	124.564	278.890	152.030	106.111	401.978	0.848

## Data Availability

The data presented in this study are available upon request from the corresponding author.
